# The WNT/ROR Pathway in Cancer: From Signaling to Therapeutic Intervention

**DOI:** 10.3390/cells10010142

**Published:** 2021-01-12

**Authors:** Kerstin Menck, Saskia Heinrichs, Cornelia Baden, Annalen Bleckmann

**Affiliations:** 1 Department of Medicine A, Hematology, Oncology, and Pneumology, University Hospital Münster, 48149 Münster, Germany; kerstin.menck@ukmuenster.de (K.M.); saskia.heinrichs@ukmuenster.de (S.H.); cornelia.baden@gmx.de (C.B.); 2 West German Cancer Center, University Hospital Münster, 48149 Münster, Germany; 3 Department of Hematology/Medical Oncology, University Medical Center Göttingen, 37099 Göttingen, Germany

**Keywords:** ROR1, ROR2, cancer, WNT signaling, non-canonical, targeted therapy, immunotherapy, CAR T cells

## Abstract

The WNT pathway is one of the major signaling cascades frequently deregulated in human cancer. While research had initially focused on signal transduction centered on β-catenin as a key effector activating a pro-tumorigenic transcriptional response, nowadays it is known that WNT ligands can also induce a multitude of β-catenin-independent cellular pathways. Traditionally, these comprise WNT/planar cell polarity (PCP) and WNT/Ca^2+^ signaling. In addition, signaling via the receptor tyrosine kinase-like orphan receptors (RORs) has gained increasing attention in cancer research due to their overexpression in a multitude of tumor entities. Active WNT/ROR signaling has been linked to processes driving tumor development and progression, such as cell proliferation, survival, invasion, or therapy resistance. In adult tissue, the RORs are largely absent, which has spiked the interest in them for targeted cancer therapy. Promising results in preclinical and initial clinical studies are beginning to unravel the great potential of such treatment approaches. In this review, we summarize seminal findings on the structure and expression of the RORs in cancer, their downstream signaling, and its output in regard to tumor cell function. Furthermore, we present the current clinical anti-ROR treatment strategies and discuss the state-of-the-art, as well as the challenges of the different approaches.

## 1. Introduction

Since the discovery of the INT1 proto-oncogene, also known as WNT1, and its identification as a key mediator of tumorigenesis, WNT signaling has evolved as one of the main pathways implicated in cancer development and progression. Nineteen different WNT glycoproteins have been identified in humans, which can potentially interact with ten members of the Frizzled (FZD) receptor family, as well as with different associated co-receptors [1]. As WNT ligands can bind to a variety of receptors, the receptor repertoire of the receiving cell, as well as the available co-receptors and downstream signaling factors, determine which downstream signaling cascade will be activated [2]. Classically, WNT signals can either trigger a β-catenin dependent, canonical or a β-catenin-independent, non-canonical signaling response. The WNT/β-catenin pathway is the best characterized WNT signaling cascade to date. It can be activated by the binding of canonical WNT ligands (e.g., WNT3A) to a FZD receptor and the co-receptor low-density lipoprotein receptor-related protein 5/6 (LRP5/6). This leads to the phosphorylation of LRP5/6 and to the association of both receptors, thereby activating Disheveled (DSH). DSH inhibits the destruction complex, consisting of adenomatous polyposis coli protein (APC), AXIN, Casein kinase 1 (CK-1), and glycogen synthase kinase-3 (GSK-3), which usually constitutively targets β-catenin for degradation. This inhibition enables the translocation of β-catenin into the nucleus where it activates the transcription of target genes predominantly involved in the regulation of cell fate, proliferation, and differentiation [3].

In addition, WNT ligands can trigger a large number of additional β-catenin-independent signaling pathways, some of which have been insufficiently analyzed. Classically, these non-canonical WNT pathways comprise the WNT/planar cell polarity (PCP) and the WNT/Ca^2+^ pathway. WNT/PCP is triggered by non-canonical WNT ligands (e.g., WNT5A) and activates the small GTPases RHOA and RAC, which are associated with the regulation of cell polarity, motility, and migration [4]. WNT/Ca^2+^, in turn, leads to the phospholipase C (PLC)-mediated release of Ca^2+^ into the cell and activates Ca^2+^-dependent effector molecules, such as protein kinase C, calcineurin, or calmodulin-dependent kinase II, which induce cellular rearrangements, as well as a transcriptional response via nuclear factor of activated associated with T cells (NFAT) [5]. Although certain WNT ligands tend to predominantly activate either canonical or non-canonical signaling, they are not exclusive for one or the other, and often can induce different signaling responses depending on the cellular context.

In recent years, it has become apparent that at least one other subpathway exists in addition to the two established non-canonical WNT subpathways, namely WNT/ROR signaling via the two receptor tyrosine kinase-like orphan receptors (RORs), ROR1 and ROR2. Both belong to the receptor tyrosine kinase (RTK) family, and although they had initially been described as orphan receptors, WNT proteins have meanwhile been identified as their long-missing ligands. Studies in mice have revealed that both RORs play a major role in development, as animals lacking ROR2 show severe developmental disorders, e.g., dwarfism, short limbs, and tails, facial abnormalities, septal defects as well as respiratory dysfunction resulting in neonatal lethality [6,7]. Although ROR1 knockout mice do not exhibit similar severe morphological disorders, they die within 24 h after birth, presumably due to respiratory dysfunction [8]. In humans, mutations in the ROR2 gene have also been described to cause two genetic disorders associated with severe skeletal defects: brachydactyly type B (BDB) and Robinow syndrome [9,10,11], which points to a central role for ROR2 in human embryogenesis as well. Mutations in ROR1 have not been linked to any human disease yet. Still, both ROR1 and ROR2 are overexpressed in many cancer entities and studies have linked overactive WNT/ROR signaling to key tumorigenic processes such as cell survival, proliferation, and invasiveness. In this review, we will summarize the current knowledge about WNT/ROR signaling in cancer, starting with the relevance of both RORs in the various tumor entities and their influence on tumor cell function. We will give a comprehensive overview about the downstream signaling elicited by ROR1 and ROR2, and revisit the current treatment strategies and first promising clinical data for targeting the WNT/ROR pathway in the context of cancer.

## 2. The ROR Family

**Figure 1:**
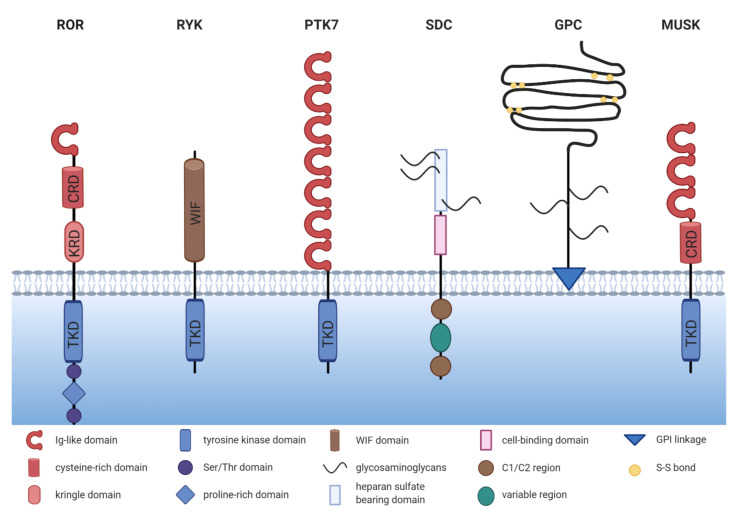
The structure of non-canonical WNT co-receptors. Comparison of the schematic structures of potential non-canonical WNT co-receptors. ROR = receptor tyrosine kinase-like orphan receptor, RYK = receptor tyrosine kinase, PTK7 = protein tyrosine kinase 7, SDC = syndecan, GPC = glypican, MUSK = muscle-specific kinase, TKD = tyrosine kinase domain, KRD = kringle domain, CRD = cysteine-rich domain.

The ROR family contains two members: ROR1 and ROR2, which are highly conserved from metazoans to humans. Both proteins share an overall amino acid identity of 58% and were already successfully cloned in 1992 from the human neuroblastoma cell line SH-SY5Y [12]. The RORs are single-pass transmembrane receptors that harbor an immunoglobulin (Ig)-like domain, a cysteine-rich domain (CRD) and a kringle domain (KRD) in their extracellular part [12] (Figure 1). The CRD of ROR1 and ROR2 is similar to that of the FZD receptors and has been identified as the essential domain for the binding of WNT ligands [13,14]. RORs are the only RTK family members possessing a KRD, which was shown to be essential for ROR1/ROR2 hetero-oligomerization [15].

The intracellular domain of the RORs exhibits a tyrosine kinase domain (TKD) and a proline-rich domain (PRD), which is framed by two serine-threonine rich domains [12]. The PRD can potentially be recognized by proteins carrying a SRC homology 3 (SH3) domain and thus mediate protein-protein-interactions. Interestingly, the PRDs of ROR1 and ROR2 show relatively low homology, suggesting that the two receptors might differ in their downstream signaling interactors. Within the intracellular domain, ROR1 has 19 tyrosines, which were shown to be phosphorylatable in silico [16]. However, the amino acid sequence of the TKD of ROR1 displays six deviations from the canonical tyrosine kinase consensus sequence, amongst which three (C482G, K614R, and L634F) are located in the catalytic center [17]. Indeed, the extent of ROR1 autophosphorylation was shown to be negligible [17,18,19]. Likewise, the structure of the TKD of ROR2 shows similarity to RTK-like pseudokinases suggesting a comparable lack of catalytic activity [20].

The RORs are predominantly expressed at the plasma membrane, although several reports have claimed a cytoplasmic, and even nuclear location of both [21,22,23,24,25,26]. The different subcellular locations might also derive from the expression of distinct isoforms, which have been described in particular for ROR1 [27]. These isoforms comprise the full-length protein expressed on the cell surface (937 aa, 100-105 kDa, unglycosylated, ROR1-001, ENSP00000360120), an intracellular/secreted variant (393 aa, ~44 kDa, ROR1-002, ENSP00000360121), and a third variant of so far unknown localization (388 aa, ~40 kDa ROR1-201, ENSP00000441637). The full-length isoform of ROR1 can be fully N-glycosylated, which results in a 130 kDa protein and permits the cell surface expression of ROR1 [28]. In chronic lymphocytic leukemia (CLL) cells a 64 kDa isoform with restricted nuclear expression as well as a 260 kDa isoform were identified, among which the latter probably represents a dimerized ROR1 (homo- or heterodimerized) [29]. The 64, 100–105, 130 and 260 kDa isoforms were shown to be phosphorylated at the tyrosine and serine residues and were differentially expressed in CLL patients, depending on their progression status [29]. Possible isoforms for ROR2 have not been described yet.

## 3. Expression of ROR1/2 in Cancer

In order to assess the targetability of ROR1 and ROR2 as novel therapeutic approach, it is important to determine their pattern and level of expression in healthy tissue, in which ROR-targeted therapies might cause undesired off-target effects, and thus toxicity. Furthermore, before choosing which cancer entities might benefit from ROR targeting, it should be noted that the RORs are not uniformly expressed in all cancer tissues, and that their function might differ in the different entities. Therefore, we discuss the current knowledge on the expression of ROR1 and ROR2 in cancer in the following chapter and summarize the findings in Table 1.

### 3.1. Expression of ROR1/2 in Healthy Tissue

Studies in mice have shown that while ROR1 and ROR2 play an important role in early embryogenesis, their expression is downregulated after birth [12,66,67,68]. The expression of ROR1 and ROR2 is not exclusive and can occur in the same cells [67,68]. In accordance with the mouse studies, in human adult tissues ROR1 was found to be expressed only at very low levels in regions of the esophagus, stomach, duodenum, colon, bladder, uterus, testis, lung, parathyroid, and primary fibroblasts, while expression was higher in pancreatic and adipose tissue [25,30,31,39,69,70]. In the hematologic system, ROR1 was discovered on immature B cells in the bone marrow, and on tonsillar B cells [32,39,71], but not on normal mature B cells, plasma cells, or peripheral blood mononuclear cells (PBMCs) from healthy donors [31,33,39,72]. Interestingly, soluble ROR1 has been detected by ELISA in the sera of healthy individuals as well as of patients suffering from CLL, albeit at low concentrations and only in <25% of cases [31]. Although the concentrations are likely too low to interfere with ROR1 antibody therapies, this observation suggests that ROR1 might undergo shedding, a finding that warrants further investigation. Among the tissues described as expressing ROR1, the protein was not only located on the cell surface, but was found in the cytoplasm in several cases [30]. While different fixation approaches were shown to affect the subcellular localization of ROR1 [22], this raises the question, whether the differences result from technical problems, or from the use of distinct ROR1 antibodies directed against the C- or N-terminus recognizing different variants of the protein.

ROR2 mRNA was absent in vital organs, but was weakly detected in the stomach, thyroid as well as in osteoblasts [57,61,73]. Furthermore, the ROR2 protein was detected in healthy colon epithelium by immunoblotting [47]. In contrast to ROR1, ROR2 was expressed on normal CD5^+^ B cells [15]. However, a comparative analysis of ROR2 protein expression in adult tissues is still lacking.

### 3.2. Expression of ROR1/2 in Hematological Malignancies

Already in 2001, gene expression analysis had identified ROR1 as an important component of a signature that distinguishes CLL cells from normal B cells, and other B cell malignancies [34,35]. The ROR1 gene was found to be 19-fold overexpressed in CLL [34]. Although the majority of CLL patients harbor high levels of ROR1-positive tumor cells in blood, ~5% of CLL cases exhibited negligible ROR1 expression [36]. ROR1 was described to increase on CLL cells upon disease progression [72], and patients with high ROR1 expression had significantly shorter therapy-free survival (TFS) as well as overall survival (OS), indicating that ROR1 is associated with a more aggressive disease [36]. ROR1 is not a unique marker for CLL, but is also highly prevalent in other non-Hodgkin lymphoma (NHL) entities, especially in mantle cell lymphoma (MCL) [37,39]. In addition, 45% of pediatric acute lymphatic leukemia (ALL) patients stained positive for ROR1 in an immunohistochemistry (IHC) study, although no association with survival was detected [30].

In contrast to the prominent role of ROR1, initial studies reported that ROR2 was not expressed in hematological malignancies, including CLL [31,72]. However, this assumption has been challenged as Yu et al. detected ROR2 on freshly isolated CLL cells and demonstrated its hetero-oligomerization with ROR1 [15]. Interestingly, ROR2 expression was lost upon longer cultivation of the cells, which might explain the differing observations. Furthermore, in multiple myeloma ROR2 was recently identified by gene expression analysis and IHC to be overexpressed on plasma cells in one third of the investigated patients [40], suggesting that further research is warranted to clarify its role in hematological malignancies.

### 3.3. Expression of ROR1 in Solid Tumors

Overexpression of ROR1 has not only been reported in hematological malignancies, but has also gained increasing attention in solid tumors. IHC analyses have demonstrated a wide-spread expression of ROR1 across many tumor entities with significantly higher expression levels in cancerous than in adjacent normal tissue (Table 1), including a particularly strong expression in melanoma, colon, pancreatic, lung, and breast cancer [22,41,56]. Chang et al. reported that high ROR1 expression was associated with a lower pathological tumor (pT) stage and the absence of perineural invasion in gastric cancer patients who underwent gastrectomy and did not receive neoadjuvant chemotherapy [50]. However, so far this remains the only analysis that suggested that ROR1 might be associated with limited disease, while the majority of studies indicated that it is rather associated with aggressiveness and poor survival. ROR1 expression was positively associated with clinical stage and lymph node metastasis in colorectal cancer, for which it served as an independent prognostic marker [46]. In ovarian cancer, high ROR1 expression was likewise associated with tumor grade as well as lymph node metastasis [58]. In Ewing sarcoma, ROR1 expression was markedly higher in metastases than in localized disease [70], and in pancreatic cancer, ROR1 was identified on circulating tumor cells (CTCs) as an essential factor for their invasive phenotype [63].

In breast cancer, ROR1 levels seem to vary between the different molecular subtypes, as gene expression data have implied particularly high levels in poorly differentiated and triple-negative breast cancers, the most aggressive breast cancer subtype, in which very high expression (top 10%) was associated with shorter OS [41]. Similar results were obtained from an IHC study in which 57% of triple-negative breast cancers stained highly positive for ROR1, whereas signals were detected in only 12% of the estrogen receptor (ER) and progesterone receptor (PR)-positive patients, and no expression at all was found in Her2/Neu-positive breast cancers [25]. Interestingly, breast cancer brain metastases have likewise been shown to highly express ROR1 [74], hinting at its role in metastatic spread.

ROR1 has also been extensively studied in lung cancer. Since it was identified as a transcriptional target of homeobox protein Nkx-2.1 [75], a major lineage-survival oncogene in lung cancer, it is not surprising that many lung tumors harbor ROR1. Comparable to breast cancer, ROR1 was expressed in certain subtypes and was particularly high in lung adenocarcinoma (40–65% positive), while squamous carcinomas stained positive at much lower frequencies [22,25,76]. In lung adenocarcinoma, ROR1 functions as an independent prognostic predictor for OS [57]. Both ROR1 and ROR2 were found to be highly expressed in a lung cancer cell line derived from CTCs from a patient with relapsed small cell lung cancer [77]. This fits to the observation in two patients with ROR1-negative primary tumors, in whom the metastases gained ROR1 [25]. While these observations suggest that ROR1 might be involved in metastasis, the same study also showed that 40% of ROR1-positive primary tumors lost their ROR1 expression in the matched metastatic lesions [25], thus questioning this concept. Taken together, this raises the question whether ROR1 solely functions as a bystander molecule upregulated during tumor progression, or whether the functional consequence of WNT/ROR signaling is highly context-specific as discussed later in this article.

### 3.4. Expression of ROR2 in Solid Tumors—It Is Not Always That Simple

Compared with ROR1, the expression profile of ROR2 in cancer is much more heterogeneous (Table 1). Although most studies describe ROR2 as a positive prognostic factor in a variety of solid tumor entities, a limited number of reports observed a loss of ROR2 in cancerous compared to normal tissue and an association of high ROR2 expression with a favorable outcome. One problem that might have caused some of the contradictory results is that several commercially available antibodies have been reported to display non-specific binding [77], thus bringing into question the reliability of the IHC results obtained with these antibodies.

A particularly high percentage of ROR2-positive tumors was found in breast cancer (87%), glioblastoma (>90%), and neuroblastoma (80%) [24,53,78]. Interestingly, ROR2 was expressed in only 20 of 48 of primary melanoma cases, while all of the investigated visceral or subcutaneous metastases (48/48) stained positive [79]. Similar observations have been reported for lymph node and brain metastases in breast cancer [74,80]. Indeed, ROR2 expression was found to correlate with tumor stage and/or lymph node metastasis in lung [55], cervical [45], and breast cancer [24]. Taken together, these studies point to a specific role for ROR2 in invasive growth and metastasis of the named tumor entities.

In contrast, downregulation of ROR2 in solid tumor cells, compared with adjacent normal tissue, has been described for gastric [52] and prostate carcinoma [65].

Contradictory findings for ROR2 have been reported for endometrial, colorectal, and ovarian cancer. Initially, the study by Lara et al. demonstrated promoter hypermethylation and consequently repressed expression of ROR2 in 5/8 colorectal cancer cell lines and 3/6 patients [47]. In contrast, another study using quantitative real-time PCR detected a significantly higher expression of ROR2 in colorectal cancer compared with adjacent normal tissue [23]. In ovarian cancer, a comparative analysis has revealed that ROR1 was largely expressed in cancer cells, while the number of patients with ROR2-positive tumors was significantly lower [81]. While ROR2 was found upregulated in a large ovarian carcinoma cohort compared with normal ovarian tissue [59], another report investigating aggressive high-grade serous ovarian carcinoma has claimed the opposite [60]. Whether these controversial findings reflect context-specific functions of ROR2, or whether they arise from non-specific antibody binding in IHC analyses remains to be clarified.

### 3.5. ROR1/2 in the Tumor Stroma

While research has traditionally focused on the expression and function of the two RORs in cancer cells, several recent reports support the notion that they also play a relevant role in the cancer-associated microenvironment. In ovarian cancer, stroma cell expression of ROR1 was lower than in the tumor cells, while the opposite was true for ROR2 [81]. Interestingly, the expression levels of ROR2 increased even further in metastatic tissue compared with matched primary tumor samples [81]. In pancreatic cancer stromal ROR2 correlated with regional lymph node invasion and represented an independent prognostic factor [64]. While the significance of stromal ROR2 remains elusive so far, novel data have shown that ROR2 is upregulated in reactive astrocytes at brain injury sites [82]. Concordantly, both ROR1 and ROR2 were induced in skeletal muscles by pro-inflammatory cytokines (e.g., TNF-α, IL-1β) released after injury [83]. These findings suggest that the upregulation of the RORs might occur in stromal cells due to the inflammatory conditions often observed in the tumor microenvironment.

## 4. The Function of ROR1/2 in Cancer

WNT signaling is known to regulate many key cellular processes, and aberrant regulation of this pathway has been found to contribute to the development and progression of many human cancers. Likewise, the deregulated expression of the WNT co-receptors ROR1 and ROR2 has been associated with several cellular features that promote malignancy, namely cell proliferation, survival, migration/invasion, and stemness, as it will be discussed in the following chapters.

While most studies identified ROR1 as an oncogene, the role of ROR2 in cancer is less clear and has been controversially discussed. Studies in osteosarcoma, melanoma, CLL, breast, and renal cancer have claimed that ROR2 acts as an oncogene in these entities. In contrast, in prostate, colorectal and endometrial cancer some studies attribute a tumor suppressive function to ROR2. Whether the output of ROR2 signaling differs especially in colorectal and endometrial cancer, two entities substantially driven by the β-catenin-dependent, canonical WNT pathway, or whether different organ microenvironments with varying repertoires of FZDs and alternative WNT co-receptors are responsible for the diverging effects is not yet fully understood.

### 4.1. ROR1/2 in Cell Proliferation and Survival

More than a decade ago both ROR1 and ROR2 were identified as pro-survival kinases by an RNAi screen in HeLa cells [84]. This finding spiked the interest of cancer biologists early on to not only evaluate the potential of these novel receptors as cancer biomarkers, but also to consider their functional involvement in tumor development and progression. Meanwhile it became evident that ROR1 plays a major role in cancer cell survival by promoting proliferation, while at the same time counteracting apoptosis. This has not only been observed in a multitude of in vitro studies on cancer cell lines [15,22,29,41,48,51,56,57,75,76], but was also confirmed in xenograft studies in vivo [41,75]. In a mouse model for human CLL, the B cell-restricted expression of ROR1 accelerated leukemogenesis through an interaction of ROR1 with the T cell leukemia 1 (TCL1) oncogene, which activated pro-survival signaling via the protein kinase AKT and resulted in leukemia cell proliferation and resistance to apoptosis [85]. In line with this, transcriptomic analyses of ROR1-expressing patient-derived CLL cells revealed a gene expression signature associated with protein kinase activation, AKT-related signaling factors and proliferation, while ROR1-negative CLL cells were characterized by subnetworks associated with apoptosis and consequential RNA processing and decay [36]. Interestingly, recent observations indicate that the pro-survival function of ROR1 not only strongly supports leukemogenesis, but is also linked with the development of leukemia. In mice, the knockout of miRNA miR-15/16, leading to the overexpression of the pro-survival factor BCL2 as well as ROR1, induced the development of B cell lymphoma in 23% of the animals, while 77% developed an aggressive acute myeloid leukemia (AML) [86]. This finding indicates a great need for further investigation of ROR1, not only in lymphoma, but also in AML, and suggests that combined targeting of ROR1 (e.g., cirmtuzumab) and BCL2 (e.g., venetoclax) might be worth considering. Taken together, these observations support the notion that the pro-survival function of ROR1 is also valid in vivo.

Mechanistic studies have shown that the effect of ROR1 on proliferation and apoptosis required phosphorylation of its PRD [16], thus suggesting an activation of ROR1-dependent downstream signaling. Concordantly, additional stimulation of ROR1 with WNT5A supported its effect on cell survival in CLL cells [33,36]. Blockade of ROR1 in lung cancer cells was found to suppress expression of CDK4 and CCNE1, two important cell cycle regulators, as well as Bcl-XL and Bcl-2, two critical anti-apoptotic factors, while it increased the expression of several pro-apoptotic factors including Bak, caspase-3, and caspase-7 [87]. However, whether these factors are direct target genes of ROR1 and which signaling pathways are implemented in their upstream regulation remains elusive so far. Moreover, two novel mechanisms that might contribute to the pro-survival function of ROR1 have recently been recognized: on the one hand, ROR1 inhibition was described to increase p53 activity [88], on the other hand, an involvement of ROR1 in autophagy was suggested [87]. Further research is required to understand the role of ROR1 in these processes in general as well as in the context of cancer.

In ovarian cancer ROR1 was described to have a synergistic effect with ROR2 on cell proliferation, since only a double knockdown led to a significant reduction in tumor cell proliferation [59]. Similar to ROR1, ROR2 supported proliferation and tumor growth, while inhibiting apoptosis both in vitro as well as in vivo in mouse models of osteosarcoma, breast and renal cancer [44,89,90,91]. Likewise, the expression of several cell cycle-related genes including CDK1/2/4, CCNE1, CCND1/2, PCNA, and MKI67 has been described to be regulated by ROR2 in these cancer entities [80,90,92]. In contrast, in a study on ovarian cancer the induction of ROR2 expression in the tumor cells led to endoplasmic reticulum stress and decreased cell viability [60].

A study in NIH3T3 fibroblasts has demonstrated that ROR2 was dynamically regulated during the cell cycle, probably because it is under the transcriptional control of the cell cycle regulator E2F1 [93]. ROR2 knockdown led to an accumulation of osteosarcoma cells in the G0/G1 phase [92] suggesting that ROR2 itself is essential for cell cycle progression. Downregulation of ROR2 not only decreased the expression of various E2F1 target genes, but also induced Forkhead box protein O1 (FoxO) target genes [93], thus favoring apoptosis induction.

Recent studies have implied that ROR1 and ROR2 might not only promote cell survival through the regulation of pro-mitotic factors, but also by controlling cellular structures implicated in pro-survival signaling, such as filopodia [28,94]. In gastric cancer cells, ROR2 was described as stimulating the number and length of specific signaling filopodia, so-called cytonemes, which transport WNT ligands to neighboring cells, and thus activate canonical WNT signaling responses stimulating cell proliferation [95]. ROR1, in contrast, was found to interact with Caveolin-1, Cavin-1, and Cavin-3 and was not only required for correct caveolae formation, but also for caveolae-dependent endocytosis and induction of signaling responses [96,97]. As several pro-survival RTKs (e.g., epidermal growth factor receptor (EGFR), hepatocyte growth factor receptor (MET), Insulin-like growth factor 1 receptor (IGF1R)) depend on these functions, inhibiting ROR1 in lung cancer was proposed as a novel shortcut to bypass resistance to EGFR tyrosine kinase inhibitors [96]. Indeed, targeting ROR1 helped to reinstall sensitivity to erlotinib treatment in resistant lung cancer cell lines [98], thus further underlining its potential as a therapeutic target.

### 4.2. ROR1/2 in Therapy Resistance and Cancer Stem Cells

Next to the established function of the RORs in cell proliferation, another detrimental consequence of active WNT/ROR signaling in cancer has currently emerged, namely the establishment of resistant tumor cell clones. Several studies have reported the upregulation of ROR1 or ROR2 in chemoresistant cancer cell lines [88,99] as well as in patient-derived tissues after chemo- or targeted therapy [100,101,102]. In melanoma, O’Connell et al. initially observed opposing roles for ROR1 and ROR2: melanoma cell lines resistant to inhibition of the serine/threonine-protein kinase B-raf (BRAF) were characterized by upregulated ROR2, but downregulated ROR1 [103]. Another study on uveal melanoma cell lines described upregulation of both RORs after mitogen-activated protein kinase kinase (MEK) inhibition and implicated them in the induction of a pro-survival AKT signaling response [104]. In ALL cell line models, and primary cells, the knockdown of ROR1 enhanced sensitivity to several small molecule inhibitors in current clinical use [105]. Addressing the question of how ROR1/2 can mediate therapy resistance, three different modes are currently being discussed:

Firstly, WNT/ROR1 signaling was identified as a rescue pathway that can induce nuclear factor kappa-light-chain-enhancer of activated B cells (NF-κB), phosphoinositide 3-kinase (PI3K)/protein kinase B (AKT), and MEK/extracellular-signal-regulated kinase (ERK) activation independent of B cell receptor (BCR)/Bruton tyrosine kinase (BTK) in CLL and MCL [18,106,107]. This activation was mediated via the formation of a complex between ROR1 and CD19 [107] and represented a novel resistance mechanism for treatment strategies centered around BTK/BCR inhibition. Indeed, synergistic effects have been shown for co-targeting both ROR1 and the BCR or BCL-2 family, underlining the large potential for ROR1-targeted therapies in overcoming MCL and CLL drug resistance [106,108].

Secondly, ROR1 was shown to upregulate the expression of ATP-dependent translocase ABCB1, a multi-drug efflux pump, and thus facilitate drug export from cancer cells [88]. Indeed, targeting ROR1 in ovarian cancer increased the efficacy of second mitochondria-derived activator of caspase (SMAC) mimetics and taxanes [109]. In line, combining paclitaxel treatment with ROR1 blockade significantly enhanced tumor growth inhibition in a patient-derived xenograft (PDX) model for breast cancer [102].

Thirdly, ROR1 has been associated with treatment-resistant cancer stem cells as reviewed in [110]. This conclusion was based on observations in ovarian cancer stem cells, in which ROR1 was highly expressed and regulated the expression of the self-renewal marker polycomb complex protein BMI-1 [111]. Meanwhile, studies on breast cancer primary cells have likewise identified a population of cells carrying the cancer stem cell markers ALDH1^+^/CD44^+^/CD24^low^ that displayed particularly high ROR1 expression and showed enhanced growth of tumor spheres in vitro as well as tumorigenicity in vivo [100,102]. These ROR1^+^ cells were characterized by enhanced stemness frequency and expressed markers typically associated with stem cells such as e.g., POU5F1, NANOG, or SOX2 [100]. Concordantly, in a phase 1 trial on cirmtuzumab, a monoclonal antibody directed against ROR1, gene expression signatures associated with stemness were markedly lower in post-treatment samples of CLL patients [112]. Taken together, these observations suggest that ROR1 is indeed expressed on cancer stem cells and might thus be involved in therapy resistance and relapse. Whether ROR2 fulfills the same functions as ROR1 in the development of drug resistance or whether it acts via distinct mechanisms remains to be elucidated.

### 4.3. ROR1/2 in EMT and Metastasis

The clinical observations that ROR1 and ROR2 are associated with disease progression and metastasis in many cancer patients have inspired researchers to investigate the role of both co-receptors in the underlying cellular processes. In CLL, stimulation of ROR1-expressing cancer cells with WNT5A stimulated their migratory potential and CXCL12/CCL19-directed chemotaxis, while this effect was not observed in ROR1-negative cells [33,36]. Similar observations have been made in breast cancer in which ROR1 knockdown decreased CXCR4 expression resulting in decreased chemotaxis of the cells towards a CXCL12 gradient [42]. Moreover, in several in vitro solid tumor models ROR1 knockdown reduced cellular migration/invasion. These include Ewing sarcoma [70], glioblastoma spheres [113], mesothelioma [57], breast cancer [42,114], ovarian cancer [59] or melanoma [56]. Another report on melanoma observed that ROR1 was associated with a poorly invasive phenotype, and its knockdown increased invasion in vitro as well as metastasis formation in vivo. However, these diverging results might be caused by the simultaneous upregulation of compensatory ROR2/WNT5A [103]. In line with cell motility, ROR1 was found to regulate cell adhesion [56].

First reports about a pro-invasive function of ROR2 stem from studies on HeLa cells showing that WNT5A-ROR2 mediated polarized cell migration in this cell line [115]. This has since been confirmed in several solid tumor entities in which knockdown of ROR2 resulted in decreased migration and/or invasion, e.g., in mesothelioma [57], melanoma [79], renal cancer [89], breast cancer [114], ovarian cancer [59,99,116,117], prostate cancer [118], leiomyosarcoma, gastrointestinal stroma tumors [62], and osteosarcoma [61]. Taken together, the data imply that both ROR1 and ROR2 are associated with highly malignant cancer cell phenotypes characterized by high motility and aggressiveness.

The pro-migratory and -invasive functions of ROR1 and ROR2 might not only result from the activation of a typical non-canonical WNT signaling response, which will be discussed in the next chapter, but have been associated with the induction of an epithelial-to-mesenchymal-transition (EMT) phenotype. EMT is a reversible cellular program in which cancer cells lose their adhesive, epithelial characteristics and gain a more motile, mesenchymal phenotype, which drives tumor spreading. ROR1-high compared to ROR1-low breast cancer tumors initially showed high expression of genes typically associated with mesenchymal cells [42]. In renal cell carcinoma patients expression of ROR2 was found to be tightly associated with the EMT regulator TWIST and the matrix-degrading enzyme MMP2 [89]. Further in vitro studies have indeed confirmed higher expression of typical epithelial factors (e.g., CK19, CDH1) and lower expression of mesenchymal factors (e.g., SNAI2, ZEB1, VIM) after ROR1 or ROR2 knockdown in cancer cells. This is indicative of a role for both co-receptors in fostering EMT [42,56,80,111,113,117]. One explanation why the RORs are so closely associated with the EMT program is that their expression has been revealed to be under the control of key EMT-inducing transcription factors. For instance, TWIST was demonstrated to activate the transcription of the ROR1 gene [119], while the expression of ROR2 was found to be regulated by SNAI1 [120]. Another report claimed that ROR2 itself was responsible for regulating SNAI1 expression [117], raising the chicken and egg question—which comes first?

The set of EMT genes regulated by ROR2, in particular, comprises the matrix metalloproteinases MMP2 and MMP9 [80,89,120]. As these enzymes are critical for degrading the extracellular matrix, their enhanced expression in ROR-positive cells might explain their large potential for invasive growth. Another mechanism by which especially ROR2 promotes invasiveness is the induction of invadopodia formation [94,121], i.e., special plasma membrane protrusions that can serve as specific sites for the secretion of matrix-degrading enzymes. Recently, ROR2 was found to upregulate the expression of intraflagellar transport 20 (IFT20), a protein originally involved in the microtubule-based transport in the cilium. IFT20 regulates Golgi structure and function which are required for correct cell polarization and subsequent invasive behavior [122]. WNT5A-ROR was also described as inducing the degradation of the kinesin KIF26b which was identified as a novel cytoskeletal effector of WNT/ROR signaling and regulated cell migration. Although the mechanism of WNT/ROR/KIF26b signaling is not fully understood yet, it was proposed that it fine-tunes de-adhesion and/or retraction at the trailing edge of migrating cells [123]. Thus, it seems that the RORs are substantially involved in regulating processes associated with cytoskeleton dynamics that finally result in increased motility and invasiveness.

Evidence suggests that these findings are also valid in vivo, as ROR1 knockdown significantly reduced the capacity of breast cancer cells intravenously injected into immunodeficient mice to invade the lung 24 to 72 h after injection [42], pointing to a role for ROR1 in extravasation and adhesion due to its control of the EMT phenotype. Similar observations have been made for ROR2: multiple myeloma cells with stable ROR2 knockdown were mostly unable to home into the bone marrow due to a defective adhesion to the bone marrow microenvironment [40]. Consequently, cancer cells with reduced expression of ROR1 or ROR2 were characterized by a significantly impaired potential in forming metastases in mouse models of melanoma or breast cancer [42,79].

However, some reports claim that in some cancer subtypes, the RORs are rather associated with less motile and less invasive tumor cells. For instance, when discussing ROR1 and EMT, the special case of hepatocellular carcinoma should be mentioned, since in that tumor entity ROR1 was associated more with an epithelial rather than a mesenchymal phenotype indicating that it might play an opposing role here [124]. For ROR2, especially in colorectal and prostate cancer conflicting data have been obtained. For the latter, ROR2 knockdown was initially found to reduce cell invasion [118]. However, a recent study reported a reduction in tumor motility and invasiveness upon ROR2 overexpression [65], again raising the question whether the regulation of ROR expression levels is critical for determining their functional impact. Moreover, WNT5A secreted by the osteoblastic niche was observed to induce dormancy in prostate cancer cells via ROR2 by repressing canonical WNT signaling and thus inhibiting bone metastasis [125]. Whether this interesting finding is specific for prostate cancer, or for bone metastasis, or if it also occurs in other tumor entities remains unknown. In the second example of colorectal carcinoma, hypermethylation, and thus silencing, of the ROR2 promoter was identified as an early event in carcinogenesis. Decreased ROR2 levels were associated with increased proliferation and migration, but with greatly reduced invasiveness [47,126]. While the early downregulation of ROR2 might represent a way to circumvent its inhibitory influence on canonical WNT signaling, the observed differential regulation of migration and invasion could indicate that ROR2 is indeed responsible for dynamically fine-tuning these cellular processes. In general, ROR2 seems to have tumor suppressive effects particularly in tumors driven by canonical WNT signaling (e.g., colorectal, prostate, or endometrial cancer), whereas it acts rather as an oncogene in tumors with predominantly active non-canonical WNT signaling (e.g., melanoma, breast cancer). This suggests that the different signaling contexts have a significant impact on the functional output of WNT/ROR signaling.

## 5. WNT/ROR Signaling at a Glance

**Figure 2:**
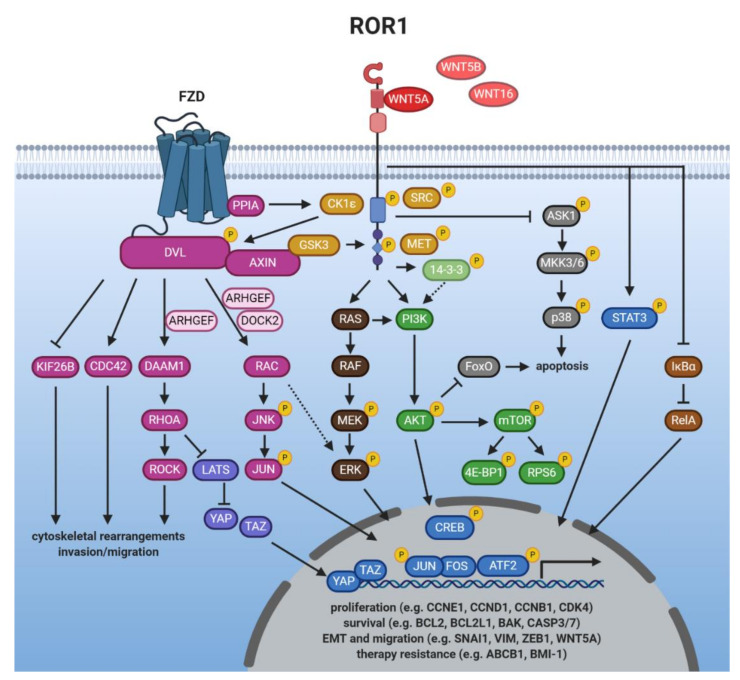
ROR1 signaling. WNT/ROR1 signaling is induced by binding of a non-canonical WNT ligand, which triggers the formation of a complex between ROR1 and ROR2, or ROR1 and a FZD receptor, respectively. Signal transduction is mediated by the phosphorylation of ROR1 by several kinases (orange) which on the one hand results in the inhibition of anti-apoptotic pathways (grey), while on the other hand activates downstream pathways such as WNT/PCP (magenta), MAPK/ERK (dark brown), PI3K/AKT (green) or NF-κB (light brown). These either trigger cytoskeletal rearrangements associated with enhanced tumor cell migration, or induce a transcriptional response (blue) leading to the expression of genes which promote cell proliferation, survival, EMT, or therapy resistance.

**Figure 3:**
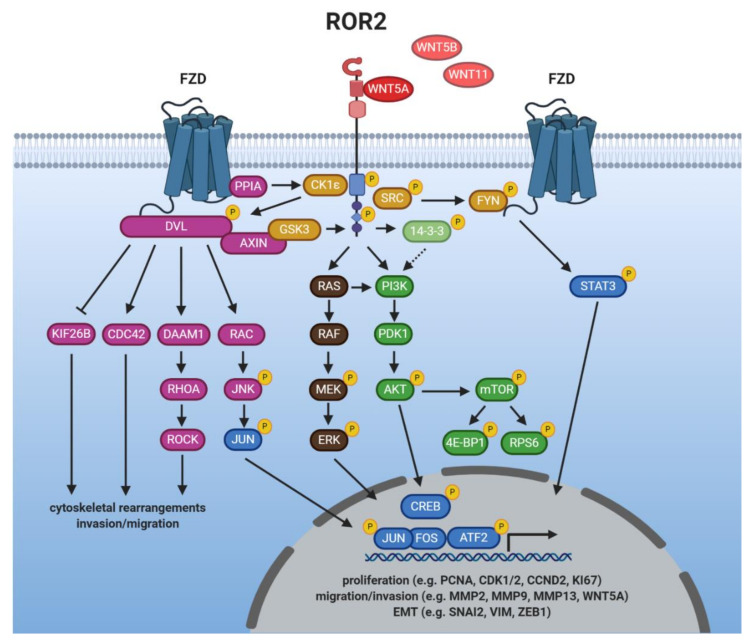
ROR2 signaling. Binding of a non-canonical WNT ligand induces the association of ROR2 with a FZD receptor (e.g., FZD2, 5, 7), or other WNT co-receptors (e.g., PTK7, ROR1). ROR2 activation is triggered by its interplay with several kinases (orange). The signal is then relayed to downstream pathways including WNT/PCP (magenta), PI3K/AKT (green), or MAPK/ERK (brown). Active WNT/ROR2 signaling results in the nuclear translocation and activation of transcription factors (blue) that mediate the expression of target genes associated with cell proliferation, survival, or EMT.

The vast majority of studies have associated both ROR1 and ROR2 with the activation of β-catenin-independent WNT signaling responses triggered by binding of the non-canonical WNT ligand WNT5A. In the following chapter, we will discuss the current knowledge about WNT/ROR signaling, which is summarized in Figure 2 and Figure 3. When modulating the expression of one of the ROR co-receptors in order to study downstream signaling events, it should be carefully considered that this could trigger upregulation of the other ROR co-receptor which might be able to compensate for the loss. For instance, in melanoma ROR1 knockdown was followed by increased expression of ROR2 and WNT5A, while the knockdown of ROR2 stimulated ROR1 expression [103], confirming a reciprocal regulation. Concordantly, in breast cancer cells ROR2 overexpression augmented ROR1 levels [114].

### 5.1. Ligands of ROR1/2

Since the two RORs were identified as harboring a CRD that resembles the WNT-binding domain found in FZD receptors, they were suspected to be able to bind WNT proteins. Indeed, both ROR1 and ROR2 have been shown to interact with the non-canonical WNT protein WNT5A [33,127] and for a long time it was thought to be the sole ligand for both receptors. However, this dogma has recently been challenged as co-immunoprecipitations in overexpressing cells have identified WNT5B and WNT16 as potential novel binding partners for ROR1 [105,128]. Regarding ROR2, it has been reported to interact with WNT5B in osteosarcoma cells [61] and preliminary results from our group indicate that WNT11 acts as a ligand for ROR2 [129]. Thus, these data suggest that the RORs display the same promiscuity in ligand binding as the FZD receptors, which adds another level of complexity to WNT/ROR signaling.

While the potential interactions with WNT5B, WNT11, and WNT16 remain to be confirmed on the endogenous level, binding of a WNT ligand alone, however, is not necessarily sufficient to trigger activation of downstream signaling. For instance, binding of WNT5A, but not WNT3A, induced phosphorylation of ROR1 or ROR2 [130,131,132]. Moreover, in contrast to WNT5A, the potential novel ROR1 ligand WNT5B failed to induce DVL3 phosphorylation [128]. In ROR2-overexpressing cells time series quantification of protein expression changes following WNT11 stimulation revealed differences in phosphoprotein networks suggesting that it might be able to trigger activation of ERK1, AKT, as well as GSK3B, known downstream effectors of ROR2 signaling [133].

Interestingly, the expression of ROR1/2 and their established ligand WNT5A is tightly associated and regulated in a positive feedback loop. ROR1 was found to upregulate WNT5A, which in turn triggered ROR1 expression via STAT3 [56,134]. Similarly, WNT5A can upregulate ROR2 levels and vice versa the downregulation of ROR2 decreased WNT5A expression [79,120,135]. A recent study has reported chemotactic migration of Ewing sarcoma cells towards WNT5A, which was impaired by ROR1 silencing [70]. Such WNT gradients might enable the secreting cells to specifically attract target cells by inducing dynamic cell movements through the regulation of non-canonical WNT/ROR signaling.

### 5.2. Getting the RORs Started: Receptor Activation

Ligand stimulation can either induce ROR1/ROR2 homo- or heterodimerization [15,136,137], or their association with FZD receptors [127,138,139]. Moreover, in HEK293 cells, ROR2, but not ROR1, was found to form a complex with PTK7 and WNT5A, which activated JNK and AP-1 [140]. Thus, it is not yet fully understood whether the RORs are indeed “true” co-receptors that are dependent on an interaction with FZDs to activate signaling, or whether they can fulfill this function alone, or via the interaction with other co-receptors (e.g., PTK7).

While the kinase function of the RORs has been a point of discussion [17,19,141], several other kinases have been implicated in their phosphorylation, and thus activation. Examples include GSK3, the receptor tyrosine kinase MET or the proto-oncogene SRC [16,17,75,130,142]. Furthermore, the WNT5A-induced association of ROR2 with FZD led to the activation of ROR2-bound CK1ε by the FZD-bound protein phosphatase 2A (PP2A) [143]. In turn, CK1ε mediated phosphorylation of ROR2 as well as FZD-associated DVL, which was required for downstream signaling [143,144]. A similar mechanism could apply for ROR1 as the phosphorylation of DVL3 was blocked upon inhibition of ROR1 and CK1 in CLL cells [145].

### 5.3. Induction of Non-Canonical WNT Signaling

DVL proteins are essential signaling hubs in the WNT pathway that become phosphorylated upon a WNT stimulus and transmit signals to downstream effectors (reviewed in [146]). This is also true for WNT/ROR signaling [56,70,91,114,121]. Activated DVL proteins can relay the signals to distinct signaling branches centered around the small GTPases RHO, RAC and CDC42 which become activated upon WNT/ROR stimulation [15,56,57,102,137]. The regulation was even observed in vivo as a gene set enrichment analysis showed that pre-treatment CLL samples displayed a higher expression of genes associated with activation of RAC1 or RHOA than matched samples after treatment with the ROR1-blocking antibody cirmtuzumab [112].

WNT/ROR-induced activation of RHOA was found to require the formin homology protein DAAM1 which interacted with DVL and recruited RHOA [147]. Next to DAAM1, several guanine nucleotide exchange factors (GEFs) are involved in the ROR-dependent activation of the small GTPases (e.g., ARHGEF, DOCK2) [15,108,148,149,150]. GEFs catalyze the exchange of free cytosolic GTP for bound GDP and are thus critical regulators of GTPase function. Concordantly, as RHO, RAC, and CDC42 control cytoskeletal reorganization, their activation explains the drastic changes in cell motility observed upon WNT/ROR signaling that result in enhanced proliferation, migration, as well as tumor engraftment in vitro and in vivo [108,148,150].

WNT/ROR signaling was furthermore shown to induce cell migration via the activation of the RAC downstream targets JNK, JUN, and ATF2 [114,115,121,127,138,151]. Although non-canonical WNT signaling has so far mainly been described to occur primarily via signaling cascades at the protein level, overexpression of ROR2 in MCF-7 breast cancer cells resulted in large changes at the transcriptomic level with more than 2860 differentially expressed genes (e.g., FAT1, VIL1, HNF4G, WIPF1). These potential novel ROR2 target genes were centered around the processes metabolism, cell remodeling and migration [152,153]. A recent study furthermore identified PLOD2, HADH, LCOR, and REEP1 as novel target genes of the ROR2/DVL2/ATF2 pathway, which were involved in the proliferation of colorectal cancer cells [154]. As so far no explicit target gene sets were known for non-canonical WNT signaling, these discoveries will facilitate further research on the signaling mediators and upstream regulators of the pathway.

### 5.4. Crosstalk with Canonical WNT Signaling

Although the WNT pathway has classically been divided into canonical and non-canonical signaling, it has nowadays become increasingly clear that this black-and-white separation is not entirely possible. There are many crosslinks at several levels, including the ROR co-receptors, in particular ROR2. While most reports did not find any indications of an interaction of ROR1 with canonical WNT ligands or with the induction of a β-catenin-dependent signaling in cancer cells [33,56,105], many reports described an inhibitory function of ROR2 on β-catenin-dependent WNT signaling [2,24,125,131,155]. Indeed, gene expression data from the TCGA database pointed to a negative correlation of ROR2 expression with an active canonical WNT signature in breast cancer patients [91], suggesting that the inhibitory effect of ROR2 also occurs in vivo. Surprisingly, other studies observed that WNT3A-induced activation of canonical WNT signaling involved ROR2 [156,157]. Taken together, it seems that ROR2 regulates the net balance of WNT signaling depending on the receptor context and the signaling factors available downstream.

### 5.5. Crosstalk with Other Major Cancer Signaling Pathways

The RORs seem to be crossroads that can link WNT signals to other major signaling pathways implicated in the regulation of key cellular processes. Among them, in particular the PI3K/AKT/mammalian target of rapamycin (mTOR) pathway seems to be closely associated with WNT/ROR signaling. Activation of ROR1 and ROR2 was shown to result in the phosphorylation of PI3K, AKT, cAMP response element-binding protein (CREB), and mTOR as well as its downstream targets S6 and 4EBP1 in numerous tumor entities [18,22,40,41,44,56,70,151,158,159]. In vivo, metastases derived from ROR1 knockdown breast cancer cells injected into immunodeficient mice showed diminished expression of phosphorylated AKT and CREB [42]. An association between ROR1, phosphorylated CREB and AKT was also observed by IHC in human gastric cancer patients [50]. In CLL patients, blockade of ROR1 by cirmtuzumab reduced mTOR-induced genes [160].

While the link between ROR1/2 and PI3K activation is not yet completely understood, ROR1 has been shown to interact with the intracellular adaptor protein 14-3-3ζ [161], which binds the p85 regulatory subunit of the PI3K complex and thus supports its translocation to the cell membrane [162]. Concordantly, ROR2 can phosphorylate 14-3-3β [136], which has been suggested as also activating PI3K/AKT signaling [163]. Thus, the interaction of both ROR receptors with proteins of the 14-3-3 family might be one mechanism by which they are able to trigger activation of the PI3K/AKT/mTOR pathway. Alternatively, in lung cancer cells ROR1 was shown to be important for sustaining the EGFR-ERBB3-mediated activation of PI3K [75], revealing another potential regulatory crosslink. Furthermore, the RORs harbor a conserved SH2 binding site in their TKD, which could mediate direct binding to PI3K.

Looking at the downstream signaling events following PI3K activation, AKT is thought to be responsible for controlling expression of EMT-related genes [164], which might explain the function of the RORs in this process. Another consequence of ROR1-mediated AKT activation is the phosphorylation, and thus inactivation, of the pro-apoptotic transcription factor FoxO [165]. Indeed, ROR1 can not only regulate FoxO activity [56,75], but was additionally shown to inhibit ASK1/p38 signaling [166]. Taken together, its crosstalk with these two pathways is probably responsible for the pro-survival effects of ROR1 observed in many cancer cells.

Next to PI3K/AKT/mTOR, there seems to be significant crosstalk of WNT/ROR1 signaling with the Hippo-YAP/TAZ pathway. Recently, the elevated levels of stress hormones observed during breast cancer progression were shown to induce glucocorticoid receptor signaling in distant tissues, which resulted in the induction of ROR1. ROR1, in turn, supported tumor colonization and metastasis formation at these sites [109]. Similar observations have been made in ovarian cancer, in which the synthetic glucocorticoid dexamethasone induced ROR1 expression, which correlated with elevated RHOA, YAP/TAZ, and BMI-1 levels in a panel of ovarian cancer cell lines as well as in the primary patient-derived cells [167]. As both YAP/TAZ and BMI-1 regulate the differentiation and self-renewing capacity of cancer stem cells, these observations could provide the molecular basis underlying the ROR1-associated stemness phenotype. The link between ROR1 and the Hippo pathway has been comprehensively reviewed in [110].

Other pathways activated by WNT/ROR signaling comprise MAPK/ERK [18,88,168], STAT3 [105,169], or NF-κB [170]. However, which specific ROR functions are mediated by the crosstalk with these pathways is not yet fully understood.

### 5.6. ROR1/2 in the Nucleus—More Than Hearsay?

Coupling of the ROR1 and ROR2 cytoplasmic domain to fluorescent fusion proteins suggested that both harbor a potential nuclear localization signal in their juxtamembrane domain and have a potential for nuclear translocation [21]. When ROR1 was forced to be expressed in the nucleus, it acted as a transcriptional modulator inducing expression of EZR, RDX, SOS, and CALD1. All four genes are associated with cytoskeletal rearrangements, and indeed, nuclear ROR1 triggered the formation of a large number of stress fibers and promoted cell migration [171]. For nuclear ROR2, no target genes have been identified, but a recent report confirmed the translocation of endogenous ROR2 into the nucleus in pancreatic ductal epithelial cells with adipocyte-derived conditioned medium containing high levels of WNT5A/B [26]. However, it remains unclear what specific role the RORs play in the nucleus and with which interaction partners they mediate their effects. Furthermore, it has to be clarified why nuclear ROR1 and/or ROR2 staining was largely absent in the IHC studies published so far. Potentially, the nuclear isoform of the RORs could undergo cleavage and thus escape detection by antibodies which are mostly directed against the extracellular domain. Alternatively, the detection of nuclear ROR2 could be caused by nonspecific binding of the antibody [77]. Further studies on endogenous ROR1/2 will have to be performed to find answers to these questions.

## 6. Targeted Therapy

**Figure 4:**
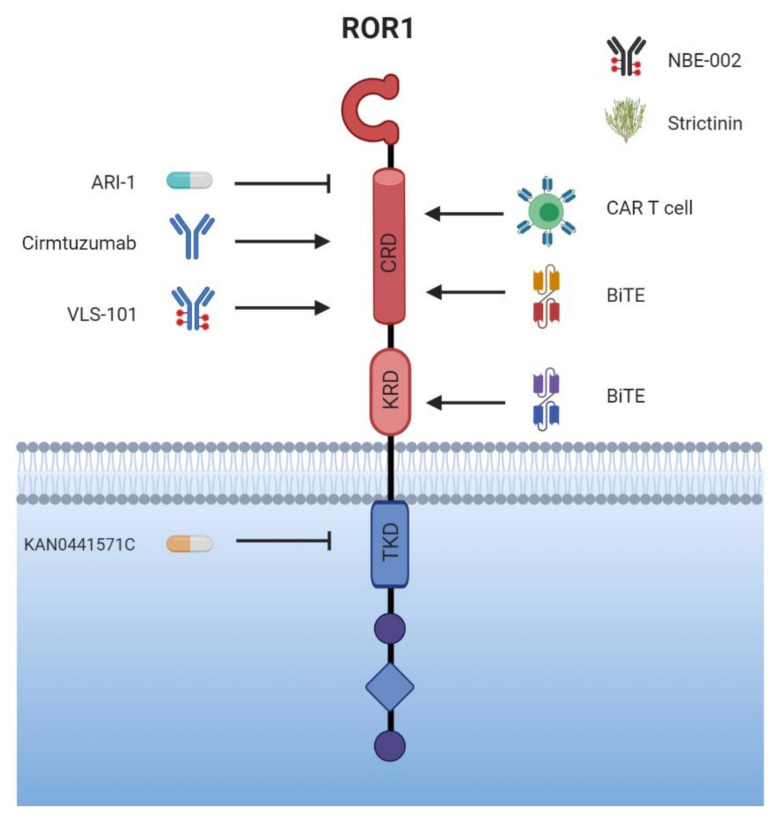
Targeting ROR1 with small molecules and immunotherapy—a selection of agents. While KAN0441571C functions as a tyrosine kinase inhibitor and already meets clinical evaluation, the inhibitor ARI-1, binding to the CRD of ROR1, is still in preclinical development. The most advanced monoclonal antibody (mAb) targeting ROR1 is cirmtuzumab. Other antibody-based targeting options comprise VLS-101, an antibody drug conjugate linking cirmtuzumab to MMAE, or bispecific antibodies such as ROR1 × CD3 or NBE-002. ROR1 is also an attractive target for chimeric antigen receptor-engineered (CAR) T cells since it does not lead to B cell depletion. Strictinin is a newly discovered, plant-derived tannin, which seemingly interacts with ROR1 and inhibits PI3K/AKT/GSK-3 activity.

The unique role of the ROR receptors as described above, being expressed primarily on cancer tissue, makes them ideal targets for therapeutic intervention. As they are not exclusively expressed on malignant tissue, worries have emerged about high toxicity of substances directed against the RORs; however, different clinical trials have so far shown good tolerability and safety. An overview of the conducted clinical trials is given in Table 2. While different approaches are currently being developed for targeting ROR1, research on ROR2 with regard to its use in cancer therapy is still in its infancy. In the following chapter, we will present the available clinical data and discuss the advances and challenges of the distinct anti-ROR treatment strategies, which are summarized in Figure 4.

### 6.1. For Starters: The Humanized Monoclonal Antibody (mAb) Cirmtuzumab

First to be mentioned in discussions about targeting ROR1 is cirmtuzumab, the first (but no longer the only) mAb binding to the extracellular domain of ROR1 with high affinity and specificity [172]. Being a competitive inhibitor for WNT ligand binding, it interrupts the signaling cascade leading to the tumorigenic effects of ROR1. Moreover, binding of cirmtuzumab induces a rapid internalization of ROR1-antibody complexes into lysosomal compartments [173]. Although ROR1 seems to be expressed in some healthy tissues as discussed above, in first preclinical studies cirmtuzumab showed no significant targeting of any post-partum healthy tissue, nor any noticeable adverse events [112]. In the past years, considerable energy has been put into evaluating the safety of cirmtuzumab in clinical trials (phase I and II) [112] in hematological B cell malignancies, where it had been identified as a tumor marker, then extending its use to ROR1-expressing solid tumors, such as breast cancer.

The first phase I trial (NCT02222688) tested cirmtuzumab in patients with relapsed or refractory CLL. It was shown to be well-tolerated, no dose-limiting toxicity was described, and only a narrow range of adverse events was published [112]. Pharmacokinetics studies showed a half-life of 32.4 days with a downregulation of ROR1 signaling-associated effects detectable for about six months, before an increase occurred. While no complete remission (CR) or partial remission (PR) was reached, 17 out of 26 patients met a stable disease situation. An affiliated extension study (NCT02860676) was performed, which tested cirmtuzumab in CLL patients for six to twelve months. The results have not yet been published. Following this lead, there were several trials with cirmtuzumab in combination with the BTK inhibitor ibrutinib in vitro and in mice, knowing that these two agents target different, but interconnected pathways associated with constitutive BCR signaling important for leukemia cell proliferation. In these preclinical experiments, the two agents showed a profitable synergy [108]. In January 2018, a phase Ib/II Study (NCT03088878) was implemented, investigating the safety and effectiveness of a combined treatment with cirmtuzumab and ibrutinib in patients with B cell lymphoid malignancies. Interim results again showed a good tolerability, no dose-limiting toxicity, and an acceptable effectiveness [174].

These results encouraged exploration in the field of solid tumors, which are similarly characterized by an overexpression of ROR1. One of the malignancies with a desperate need for new and targeted therapies is breast cancer. High ROR1 levels in breast cancer are associated with chemoresistance, metastasis, and poorer outcomes [41,102]. A preclinical study has demonstrated that cirmtuzumab infusions every two weeks inhibited re-engraftment of breast cancer cells and the formation of pulmonary metastases in a mouse PDX model. Evaluating the combination of chemotherapy with paclitaxel and immunotherapy with cirmtuzumab showed the great benefit in combining the two [102] as discussed in Section 4.2. A phase Ib trial of cirmtuzumab in combination with paclitaxel was therefore initiated for the treatment of patients with metastatic, or locally advanced, unresectable breast cancer (NCT02776917). Although the completion date is anticipated in 2021, first preliminary findings seem promising [175].

### 6.2. New Approaches—Going beyond Cirmtuzumab?

In addition to cirmtuzumab, a second generation mAb targeting ROR1 has been created: 5F1-B10, which induced apoptosis in melanoma and bladder cancer cells by inhibiting pro-survival ROR1 signaling [29,176]. Apart from its potential in cancer therapy, it has been furthermore suggested as a diagnostic tool for detecting bladder cancer cells by flow cytometry in urine samples of patients. However, as cirmtuzumab is a well-developed, already (getting) established agent that has gained a foothold, catching up with it will be surely a challenge to overcome.

An alternative approach in cancer immunotherapy is therapeutic cancer vaccination. In a preclinical study by Wu et al. a vaccine based on ROR1-expressing ovarian cancer stem cells induced high immunogenicity and prophylactic effectiveness against ovarian cancer [177]. Although ROR1 could indeed serve as a promising tumor antigen with high immunogenicity, therapeutic cancer vaccines still face the challenges of overcoming cancer immunosuppression and eliciting an adequate immune response; major obstacles that have thus far resulted in disappointing results of the vaccination approach in patients.

### 6.3. More Is More: Conjugates and Bispecific Antibodies

Besides mAbs, there are many more therapeutic options currently being developed based on the activation of the humoral immune response to counteract tumor growth, namely conjugates and bispecific antibodies. Bispecific antibodies (biAbs) are molecules combining a constant region Fc- and an antigen-binding Fab-fragment derived from two different mAbs (first generation biAb), or second generation biABs that combine two single chain variable fragments (scFv) from different antibodies. While one of the scFv connects to a target cell, the other one binds a T cell, which is then activated. Such bispecific T cell engagers (BiTEs) represent a subclass of biAbs, defined by a CD3-specific arm and a tumor cell-specific arm [178]. Associated with this intended mechanism of action are a range of severe adverse events, such as the cytokine-release-syndrome (known also from the application of chimeric antigen receptor-engineered (CAR) T cells). Qi et al. designed a bispecific ROR1 × CD3 scFv-Fc format binding to a membrane-proximal epitope of the KRD [179]. Using in vitro and in vivo approaches, they showed it to be more active than biAbs binding to a membrane-distal epitope of ROR1 since it facilitated the formation of the cytotoxic immunological synapse and receptor clustering [180]. Interestingly, BiTEs showed superior cytotoxicity against various tumor cell lines when composed of scFvs directed against the CRD as opposed to against the Ig-like domain [181]. Although no T-cell activation was observed in ROR1-negative tissue, the anti-tumor activity of the BiTEs did not depend on the actual level of ROR1 expression, demonstrating a low effector to target ratio. Nonetheless, lower drug doses were required compared with conventional mAbs, which argues for a higher potency of biAbs in inducing an immune reaction. Compared with CAR T cell therapy, which will be discussed below, continuous drug infusion is necessary for antibody treatment because of the drug clearance. The upside of this is that its action can be rapidly terminated at any time in the case of an adverse event by simply stopping its administration. This is in contrast to the uncontrollable expansion of CAR T cells in vivo [181,182].

Since it had already been in clinical testing, it seemed advantageous to use cirmtuzumab to create an antibody-drug conjugate (ADC) by linking it to the anti-neoplastic agent monomethyl auristatin E (MMAE). Based on cirmtuzumab’s high binding specificity towards ROR1-positive cells and its rapid internalization into target cells, this approach promises to safely deliver and release MMAE into the tumor cell [173,183]. Two such ADCs were developed in parallel, VLS-101 and cirmtuzumab—ADC-7 [173,184]. Cirmtuzumab—ADC-7 was observed to cause a CR of ROR1-positive CLL and MCL cells in animals. Moreover, the combination of venetoclax with cirmtuzumab—ADC-7 was tested and showed a valuable synergistic effect in targeting ROR1-positive leukemia and lymphoma cell lines. Concordantly, in a PDX model for Richter’s syndrome, a rare complication of CLL resulting in transformation towards an even more aggressive form of lymphoma, VLS-101 demonstrated a high specificity towards ROR1 by reaching tumor cells in peripheral blood, bone marrow, and spleen, leading to significant disease regression. CR was even observed in lymphoma cells without universal ROR1 expression, an effect that was attributed to the high toxicity of MMAE. No further tumor growth was documented even several months after the end of treatment, which led to longer patient survival [184]. Currently, VLS-101 is being tested in a phase I clinical trial (NCT03833180).

An alternative ROR1-based ADCs is NBE-002, a conjugate of a mAb targeting ROR1 and the anthracycline PNU-159682 (a bioactive agent of nemorubicin). First preliminary results indicated that the conjugate showed cytotoxicity in various solid tumor entities, with the strongest effect observed in triple-negative breast cancer cells [185]. A tumor rechallenge in formerly NBE-002-treated, tumor-free animals resulted in an inhibition of tumor growth, although NBE-002 was not applied again, implying that an immunological memory was being triggered. A combination with checkpoint inhibitors also showed promising results in a preclinical setting [185]. First clinical results are to be announced in 2021. For CLL, MCL, and pre-B-ALL cells, the anti-ROR1 antibody drug conjugate huXBR1-402-PNU is currently being evaluated pre-clinically in vitro and in vivo [186]. Considering that ADCs such as, e.g., brentuximab vedotin or ado-trastuzumab emtansine are nowadays successfully used in the clinic for treating specific cancer entities, the strategy of also using anti-ROR1 ADCs to target ROR1-positive tumors seems especially promising.

### 6.4. CAR-T Cells—Promising Future or More about Adverse Events?

Another way of inducing activation of the humoral immune system in antineoplastic therapy is with CAR T cells. These are autologous T cells engineered to carry receptors specific for tumor antigens, e.g., CD19 or ROR1, thus directing the T cell towards the tumor cell. Following binding, the engineered T cell gets activated, focusing its cytotoxic activity on the malignant cell. Since CAR T cells are able to proliferate, the antineoplastic effect is deemed to be persistent for a longer period of time, consequently as are the adverse events. Up to date, off-target cytotoxicity represents one of the main challenges in CAR T cell therapy, among other reasons because it requires a suitable antigen with a high selectivity towards malignant cells [187]. The success of CAR T cell therapy does not only depend on the design of the CAR T cell itself though, but also on the tumor burden, the former lymphodepletion, and the type of malignancy (solid or hematologic) to be targeted. Until now, CAR T cell therapy in solid tumors is not as effective as in hematological malignancies. A number of reasons contribute to this circumstance, one of them being a lower specificity towards the target cell. This could be enhanced by engineering a CAR T cell that recognizes two antigens on one target cell, thus fostering T cell activation. Other reasons are the mechanical and biochemical obstacles the CAR T cells have to overcome in order to reach their target tumor cell within the tissue. It has been shown that CAR T cells in solid tumors run down quicker, expressing signs of exhaustion earlier than the equivalents in hematological malignancies due to the immunosuppressive microenvironment that the tumor creates [188,189].

All in all, ROR1 CAR T cells achieved promising preclinical results. The effectiveness of ROR1 CAR T cell therapy was reported in three-dimensional (3D) cell culture models of lung and breast cancer, however, it was also documented that the CAR T cells showed signs of exhaustion already after a short period of time. Whether this disadvantage can be overcome by a second boost infusion remains to be tested [190]. ROR1 CAR T cells also showed effectiveness in high risk sarcoma xenograft models (e.g., Ewing sarcoma, osteosarcoma, rhabdomyosarcoma) [191]. As already mentioned, one concern when applying ROR1-directed CAR T cells is potential off-target activity, and thus toxicity. Although ROR1 is generally not expressed in healthy tissue, it is detected at higher levels in adipocytes and pancreatic cells. Indeed, incubation of ROR1 CAR T cells with primary adipocytes in vitro resulted in T cell activation and the release of Interleukin-2, Interferon-γ, and granulocyte macrophage colony-stimulating factor, pointing to potential cross-reactivity [25,39]. However, no toxicity was reported after infusion of CAR T cells in macaques, who have a similar ROR1 distribution and expression pattern in normal tissues as humans [25], even after the infusion of very high doses of functional ROR1 CAR T cells [69]. Thus, the potential cross-reactivity seems neglectable in vivo. These efforts and the promising preclinical data led to the implementation of a phase I study with ROR1 CAR T cells (NCT02706392), which does not only include patients with hematological malignancies, but also solid tumors. The projected primary completion date is December 2021.

### 6.5. Small Molecule—Big Impact?

A different approach in targeted therapy are small molecule inhibitors. Recently, the first anti-ROR1 inhibitor was designed which prevented phosphorylation of the TKD and thus blocked ROR1-induced signaling [192]. In vitro, treatment with KAN0439834 induced apoptosis of ROR1-positive CLL cells. The EC50 was reported to be over 60-fold higher for ROR1-positive than -negative cells, and neither normal T nor B cells were targeted by the compound. Compared with the anti-tumor effects of other inhibitors commonly applied in the treatment of CLL, KAN0439834 reached similar levels of apoptosis induction as venetoclax (Bcl-2 inhibitor) in patient-derived CLL cells, and was more effective than ibrutinib (BTK inhibitor) and idelalisib (PI3K inhibitor). In a direct comparison with venetoclax, KAN0439834 showed a higher specificity for CLL cells and displayed less cytotoxicity for healthy PBMCs [192]. Given the overexpression of ROR1 in many solid tumors and the urgent need for targeted strategies for these entities, as well, KAN0439834 has already been tested for application in pancreatic cancer [193]. In this preclinical study on pancreatic cancer cell lines, it proved equally effective in inducing tumor cell apoptosis, at even lower EC50 values than with erlotinib (EGFR inhibitor) or ibrutinib. Interestingly, KAN0439834 was reported to induce significantly higher levels of apoptosis than a ROR1-targeting mAb. However, whether this difference holds true in direct comparison with the established ROR1 mAb, cirmtuzumab, remains to be clarified. On a molecular level, KAN0439834 inhibited the phosphorylation of PI3K/AKT/mTOR/CREB and SRC, both in CLL as well as in pancreatic cancer cells [192,193], and thus blocked major pro-survival pathways induced by ROR1. As these pathways can also be activated by EGFR, which is frequently overexpressed in pancreatic cancer, the combination of KAN0439834 with erlotinib or ibrutinib (which can also function as an EGFR inhibitor [194]) showed clear additive effects and highlighted the benefit of potential combinatory treatment strategies [193].

Based on these promising results, an improved second-generation small molecule inhibitor (KAN0441571C) was developed. It has so far only been tested in diffuse large B cell lymphoma cell lines. KAN0441571C showed a higher cytotoxicity than KAN0439834, impressing with a longer halftime in mice than its predecessor (11 h instead of 1.2 h), although there was no improvement in terms of specificity towards ROR1. Inhibition of downstream ROR1 pro-survival signaling was observed as with KAN0439834 and resulted in dose-dependent cytotoxicity. In cell viability assays KAN0441571C was superior to ibrutinib, but showed similar cytotoxicity as venetoclax [38]. However, the combination of KAN0441571C with the latter resulted in almost complete tumor cell killing, again underlining the benefit of combined treatment. Picking up on these developments, another small molecule inhibitor was designed (ARI-1), which directly targets the extracellular WNT-binding CRD of ROR1 [195]. Treatment with ARI-1 blocked downstream ROR1 signaling via PI3K/AKT/mTOR, thus inhibiting proliferation and migration of ROR1-positive non-small cell lung cancer cells in vitro and in vivo. ARI-1 was reported to show a high specificity for ROR1 and was effective also in cells that harbored resistance against EGFR-targeting substances. This fits to the report discussed earlier that, in lung cancer cells, targeting ROR1 can help to circumvent resistance due to its role in sustaining signaling of several pro-survival RTKs [96]. The observation that ARI-1 did not cause severe toxicity towards the heart, liver, spleen, lung, or kidney in a mouse xenograft model is promising for its possible application in human patients [195]. Since the conducted studies have so far been limited to preclinical evaluation, clinical data will be required to determine the potential and the limitations of the developed small molecule inhibitors with regard to the development of resistance mechanisms, which is a common drawback in this therapy approach. Skipping to triple-negative breast cancer, a South African plant has come into the focus of ROR1 targeting strategies: *Myrothamnus flabellifolius* contains strictinin, a polyphenol which appears to be a competitive ligand for ROR1 binding sites and can therefore inhibit ROR1 signaling in the tumor cells. However, in silico experiments show that a high expression of ROR1 is needed for a therapeutic effect [196].

### 6.6. ROR2, the Wallflower?—To Be Continued

When it comes to ROR2, the degree of its translation into the clinic does not yet match that of ROR1. However, based on the functional data presented in the previous chapters, this seems surprising since evidence has accumulated for several solid tumors that clearly highlight ROR2 as an oncogene in these entities (e.g., breast cancer, lung cancer, and sarcoma). The current ROR2-centered research activities comprise mostly adoptive immunotherapy approaches, as the kinase activity of ROR2 is still a matter of debate. What is known, though, is that the TKD of ROR2 is relatively unique amongst the RTK superfamily [197], and could be exploited in the development of small molecule inhibitors. Ongoing research on targeting ROR2 by adoptive immunotherapy includes mAbs, CAR T cells, and ADCs. Peng et al. designed the rabbit mAb XBR2-401, which binds to a membrane-proximal epitope in the KRD of ROR2 [198]. Fortunately, the extracellular domains of human and rabbit ROR2 are very similar which promises valuable preclinical results. Subsequently, they developed the mAb into a CAR T cell format, which demonstrated a high specificity towards ROR2. Moreover, XBR2-401 was used as a base for designing a biAb (ROR2 x CD3), showing specificity for ROR2 in vitro [199]. Further investigation concerning ROR2-targeting ADCs are also in progress [200]. One potential candidate might be BA3021, a CAB-ROR2-ADC, which reversibly interacts with ROR2 in conditions reflecting the tumor microenvironment, but less so in normal tissue [201]. It is currently being tested in a clinical phase I/II study in patients with advanced solid tumors (NCT03504488). When looking at the clinical trials registered to date, there are two additional studies investigating the ROR2-specific CAR T cells in solid malignancies expressing ROR2 (NCT03960060) or, more specifically, ROR2-positive renal carcinomas (NCT03393936). However, neither are yet recruiting; therefore, the clinical success of these treatment strategies remains to be seen.

## 7. Conclusions

Starting with the identification of the RORs as orphan receptors, it is now clear that both ROR1 and ROR2 are essential members of the WNT pathway that can bind WNT ligands and activate downstream β-catenin-independent signaling. While ongoing research has begun to reveal the pro-tumorigenic functions of ROR1 and ROR2 in cancer, the mechanisms underlying their regulation and their context-dependent functionality in the distinct tumor entities, which has caused conflicting observations, are still largely unknown. Nonetheless, both are attractive targets for targeted therapy in selected tumor types, especially in combination with established drugs (e.g., erlotinib, venetoclax, ibrutinib) or even with anti-CD19 CAR T cell therapy. Given the current lack of possibilities to clinically target non-canonical WNT signaling despite its increasingly acknowledged relevance in human cancer, the ongoing development of anti-ROR therapy strategies is surely exciting.

## Figures and Tables

**Table 1:** Expression of ROR1 and ROR2 in cancer.

Cancer Entity	Expression ^1^	Correlation with Survival	Reference
**Hematological Malignancies**			
acute lymphocytic leukemia	ROR1 +	no correlation found	[18,30]
chronic lymphocytic leukemia	ROR1 +	high ROR1 correlates with poor OS and TFS	[31,32,33,34,35,36]
diffuse large B cell lymphoma	ROR1 +	n.d.	[37,38]
follicular lymphoma	ROR1 +	n.d.	[37,38]
mantle cell lymphoma	ROR1 +	n.d.	[37,39]
marginal zone lymphoma	ROR1 +	n.d.	[37]
multiple myeloma	ROR2 +	n.d.	[40]
**Solid Tumors**			
breast cancer	ROR1 +	high ROR1 correlated with poor OS, MFS and DFS	[41,42,43]
	ROR2 +	high ROR2 correlated with poor DFS	[44]
cervical cancer	ROR2 +	high ROR2 correlated with poor OS and RFS	[45]
colorectal cancer	ROR1 +	high ROR1 correlated with poor OS	[46]
	ROR2 +/−	high ROR2 correlated with poor OS	[23,47]
endometrial cancer	ROR1 +	high ROR1 correlated with poor OS and PFS	[48,49]
	ROR2 +	no correlation found for ROR2	
gastric cancer	ROR1 +	no correlation found for ROR1	[50,51]
	ROR2 −	n.d.	[52]
glioblastoma	ROR2 +	no correlation found	[53]
lung cancer	ROR1 +	high ROR1 correlated with poor OS	[22,54]
	ROR2 +	high ROR2 correlated with poor OS	[55]
melanoma	ROR1 +	high ROR1 correlated with poor PRS	[56]
mesothelioma	ROR1 +	n.d.	[57]
	ROR2 +		
ovarian cancer	ROR1 +	high ROR1 correlated with poor OS and DFS	[58]
	ROR2 +/−	no correlation found for ROR2	[59,60]
sarcoma	ROR2 +	high ROR2 correlated with poor OS in GIST high ROR2 correlated with poor DSS in leiomyosarcoma	[61,62]
pancreatic cancer	ROR1 +	n.d.	[63]
	ROR2 +	high ROR2 correlated with poor OS	[64]
prostate cancer	ROR2 −	n.d.	[65]

^1^ +/− = higher/lower expression compared to normal tissue; n.d. = not determined, OS = overall survival, TFS = therapy-free survival, MFS = metastasis-free survival, DFS = disease-free survival, RFS = recurrence-free survival, PFS = progression-free survival, PRS = post recurrence survival, DSS = disease-specific survival, GIST = gastrointestinal stromal tumor.

**Table 2:** Clinical trials with ROR1- and ROR2-targeting therapies.

Study	Clinicaltrials.gov Identifier	Regimen	Disease	Phase of Development	Estimated Study Completion Date	Status	Sponsor
UC-961 (Cirmtuzumab) in Relapsed or Refractory CLL	NCT02222688	cirmtuzumab	CLL	I	October 2017	completed	University of California, San Diego
An Extension Study of UC-961 (Cirmtuzumab) for Patients With CLL Treated Previously With UC-961	NCT02860676	cirmtuzumab	CLL	I	February 2018	completed	University of California, San Diego
A Study of Cirmtuzumab and Paclitaxel for Metastatic or Locally Advanced, Unresectable Breast Cancer	NCT02776917	cirmtuzumab + paclitaxel	breast cancer	Ib	June 2021	recruiting	University of California, San Diego
A Study of Cirmtuzumab and Ibrutinib in Patients With B-Cell Lymphoid Malignancies	NCT03088878	cirmtuzumab + ibrutinib	B-CLL MCL SLL	I/II	December 2022	recruiting	University of California, San Diego
A Study of VLS-101 in Patients With Solid Tumors	NCT04504916	VLS-101	lung cancer breast cancer	II	July 2022	recruiting	VelosBio Inc.
A Phase 1 Dose-Escalation and Cohort-Expansion of VLS-101 in Hematologic Malignancies	NCT03833180	VLS-101	hematologic malignancies	I	June 2021	recruiting	VelosBio Inc.
NBE-002 in Patients With Advanced Solid Tumors	NCT04441099	NBE-002	breast cancer and other solid tumors	I/II	July 2023	recruiting	NBE-Therapeutics AG
CAB-ROR2-ADC Safety and Efficacy Study in Patients With Solid Tumors	NCT03504488	CAB-ROR2-ADC	lung cancer breast cancer sarcoma and other solid tumors	I/II	May 2022	recruiting	BioAtla, LLC
Genetically Modified T-Cell Therapy in Treating Patients With Advanced ROR1+ Malignancies	NCT02706392	ROR1 CAR T cells	lung cancer breast cancer ALL MCL CLL	I	December 2021	recruiting	Fred Hutchinson Cancer Research Center, Seattle
Safety and Efficacy of CCT301 CAR-T in Adult Subjects With Recurrent or Refractory Stage IV Renal Cell Carcinoma	NCT03393936	ROR2 CAR T cells	renal cell carcinoma	I/II	January 2021	not yet recruiting	Shanghai PerHum Therapeutics Co., Ltd.
A Study of CCT301-59 CAR T Therapy in Adult Subjects With Recurrent or Refractory Solid Tumors	NCT03960060	ROR2 CAR T cells	sarcoma gastric cancer pancreatic cancer bladder cancer and other solid tumors	I	February 2021	not yet recruiting	Shanghai PerHum Therapeutics Co., Ltd.

ALL = acute lymphocytic leukemia, CLL = chronic chronic lymphocytic leukemia, MCL = mantle cell lymphoma, SLL = small lymphocytic lymphoma, ADC = antibody-drug conjugate, CAR = chimeric antigen receptor.

## Data Availability

No new data were created or analyzed in this study. Data sharing is not applicable to this article.
